# Association between hair cortisol concentration and dietary intake among normal weight preschool children predisposed to overweight and obesity

**DOI:** 10.1371/journal.pone.0213573

**Published:** 2019-03-08

**Authors:** Sofus C. Larsen, Jeanett F. Rohde, Nanna J. Olsen, Mina N. Händel, Maria Stougaard, Jan Fahrenkrug, Berit L. Heitmann

**Affiliations:** 1 Research Unit for Dietary Studies, The Parker Institute, Bispebjerg and Frederiksberg Hospital, The Capital Region, Frederiksberg, Denmark; 2 Department of Research and Development, Health Science, University College UCC, Hillerød, Denmark; 3 Department of Clinical Biochemistry, Faculty of Health Sciences, Bispebjerg Hospital, Copenhagen NV, Denmark; 4 The National Institute of Public Health, University of Southern Denmark, Copenhagen, Denmark; 5 The Boden Institute of Obesity, Nutrition, Exercise & Eating Disorders, The University of Sydney, Sydney, Australia; 6 Department of Public Health, University of Copenhagen, Copenhagen, Denmark; Vanderbilt University, UNITED STATES

## Abstract

**Background:**

The association between chronically elevated cortisol, as measured by hair cortisol concentration (HCC), and dietary intake among children has generally not been explored. Moreover, it is unknown whether there is an association between parental HCC and dietary intake among their children.

**Objective:**

To examine associations between HCC and dietary intake among children, and to explore the association between parental HCC and dietary intake among their children.

**Methods:**

We conducted a cross-sectional study based on 296 children predisposed to overweight and obesity who participated in the Healthy Start study. Multiple Linear regression analyses were conducted to assess the association between HCC and total energy intake, macronutrients, fruit and vegetables, added sugar, sugar-sweetened beverages (SSB), and a diet quality index (DQI).

**Results:**

Among the children, we found that higher HCC was associated with a lower consumption of dietary fat (β: -0.7 g/day [95% CI: -1.3, -0.0] per 100 pg/mg HCC). We found no statistically significant association between HCC and intake of total energy, protein, carbohydrate, fruit and vegetables, added sugar, SSB or DQI. We found no association between parental HCC and intake of total energy, added sugar, selected food groups or DQI among their children. However, stratified analyses showed that paternal HCC was associated with a borderline significant lower total energy intake and significantly lower protein intake, but only among daughters (adjusted β: -42 kcal/day [95% CI: -85, 0] and -2.6 g/day [95% CI: -4.4, -0.8] per 100 pg/mg HCC, respectively).

**Conclusion:**

Among children, chronic stress as measured by HCC may be associated with a lower fat consumption, and paternal HCC may be associated with a lower intake of energy and protein among their daughters. However, the associations observed were weak, and any clinical relevance of these findings remains questionable.

## Introduction

Several studies have shown a relationship between stress and obesity among children and adults [1–4], and that parental stress is associated with obesity in their children [5]. The underlying causes of these associations are not fully understood, but it is well known that both psychosocial stress and obesity are linked to an unhealthy lifestyle [6]. In this regard, a number of studies have suggested that psychosocial stress, as measured by questionnaire, is associated with a less healthy diet and higher intake of fatty and sweet foods [7–10]. Moreover, as parents have a high degree of influence on what their children eat, a relationship between parental stress levels and their children's eating habits is also likely, and a direct association between parental perceived stress and higher fast food consumption among their children has been reported [5]. Hence, both child and parental stress level may be a barrier for making healthy dietary choices necessary for long term weight control. However, the relationships may not be straight forward, as it has also been suggested that while some individuals have a propensity to increase their caloric intake during periods of stress, others tend to reduce their caloric intake [11].

The Hypothalamic-Pituitary-Adrenocortical (HPA) axis is a well-known pathway in stress response, and may play a role in the link between stress and dietary intake [12]. HPA axis activation begins in the hypothalamus and goes via the pituitary to the adrenal glands, leading to an increased cortisol secretion in the blood. Cortisol measured in blood, saliva or urine has been the most used biological measures of stress, and results from a cross-sectional study of 323 Belgian children (5–10 years of age) suggested that higher saliva cortisol concentrations were associated with a higher consumption of fatty and sweet foods [13]. However, a limitation of cortisol measured in blood, saliva or urine is that these measures only provide information of acute cortisol excretion, thereby expressing acute rather than chronic stress [14].

Hair cortisol concentration (HCC) is a relatively new biomarker reflecting activity of the HPA axis during the period of hair growth, thus providing a biological measure of long-term cortisol exposure [15]. Several studies have suggested a direct relationship between HCC and adiposity among children and adults [14,16–23], and we have shown that a higher maternal HCC is associated with a higher fat mass and a lower fat free mass in their offspring [14]. It seems plausible, that these associations could be mediated by dietary factors, but studies have generally not explored association between HCC and dietary intake. Moreover, the majority of studies investigating the relationship between stress and dietary intake have been performed in adult populations.

Thus, to build on the current evidence, suggesting a direct link between stress and obesogenic diet among children, we examined associations between HCC and total energy intake, macronutrients, food groups and over all diet quality in a group of preschool children predisposed to obesity. Furthermore, we explored the association between parental HCC and dietary intake among their children.

## Methods

### Study population

The study was based on data from the Healthy Start project, which was registered at ClinicalTrials.gov (ID: NCT01583335). The Danish Data Protection Agency approved the study (present: no. 2015-41-3937; previous: no: 2007-41-0530). The Scientific Ethical Committee of the Capital Region in Denmark decided that according to Section 2.-(1) of the Danish Act on a Bioethics Committee System and the Processing of Bioethics Projects, the project was defined not to be a bioethics project and as a result did not need approval from the Danish Bioethics Committee (journal number H-A-2007-0019). Written informed consent to use the collected data for research purpose was obtained from all participants’ parents [24]. The study included children who were born in 11 municipalities from the greater Copenhagen area, were aged 2–6 years at baseline, and were primarily normal weight, but at high risk of future obesity. Based on information from the Danish Medical Birth Registry and administrative birth forms, children were considered at high risk of obesity and selected if they either had a high birth weight (> 4000 gr.) or an overweight mother (BMI ≥ 28 kg/m^2^). Furthermore, a subgroup from one municipality was selected based on having a mother with low socioeconomic status (≤ 10 years of education). The intervention took place over on average 15 months in the period between 2009 and 2011 and consisted of individual guidance to optimizing dietary and physical activity habits, reducing chronic stress and stressful events and improving sleep quality and quantity. The intervention was family-based rather than solely targeting the individual child. As HCC was only measured at the follow-up examination, the present study is cross-sectional and based on follow-up information from children in the intervention group (n = 320) and the control group (n = 315), and their parents. HCC was measured in the children and their parents, information on dietary intake of the children was collected, and information on lifestyle factors was obtained by questionnaires [25]. HCC measurements were available from a total of 364 children, 335 mothers, and 251 fathers. On these individuals, information was available on the child’s dietary intake and covariates on a total of 296 children, 272 of the mothers and 208 of the fathers (S1 Fig). A total of 193 children had both their parents included in the present study. Information on number of participants enrolled based on each inclusion criteria can be found in S2 Fig.

### Hair cortisol concentration

The concentration of cortisol in hair samples was determined by a modification of a previously described protocol [26]. Hair samples were cut from the posterior vertex as close to the scalp as possible by the health counsellors. If both parents were not present at the consultation, instructions were given on how to obtain hair samples at home. The samples were stored in aluminium foil, and the scalp end of the hair sample was carefully marked. Between 10 and 20 mg of hair from the 1–2 cm closest to the scalp was accurately weighed and minced finely with scissors. One milliliter of methanol was added and the suspension was incubated overnight at 50°C with a gentle shaking. The following day, the methanol was transferred into a clean tube and evaporated to dryness under nitrogen. The residue was reconstituted in 250 μl PBS buffer (pH 8.0). The cortisol concentration in the resulting buffer solution was determined in duplicate using a commercially available salivary cortisol enzyme-linked immunosorbent assay (ALPCO Diagnostics, Salem, NH, USA). Twenty-seven assays were conducted with an 8.0% intra-assay coefficient of variation. The assay sensitivity was 16.7 pg/mg based on a hair mass of 15 mg. The reproducibility of the assay determined by analysis of aliquots of the same hair samples in different assays was 15% [14,27]. Hair cortisol concentration was included in the statistical analysis in units of pg/mg.

### Dietary intake

At baseline and follow-up, the parents were asked to fill out a four-day dietary record from Wednesday to Saturday on behalf of their children. To help the families estimate portion sizes, the dietary records were supplemented with a picture book including seventeen series with foods and portion sizes. We used Dankost 3000 to calculated nutritional composition of the participants’ dietary intake (http://dankost.dk). This software is based on the official Danish food composition database (version 7.01) [28]. Dietary intake of total energy, total fat, saturated fat, carbohydrate, added sugar and protein was included in the analyses in units of kcal/day or g/day and energy percentage (E%).

Since it was not possible to define food groups, using the software Dankost 3000, this was done manually by studying the list of all food items consumed by the children. A Stata program was then developed to extract information and generate food groups. For the present study, consumption of sugar-sweetened beverages (SSB) (including soft drinks, squash [concentrated fruit syrup added to water], chocolate milk, milkshake and drinking yoghurt), was reported by participants as number of standard glasses for children (150 ml) and included in the analyses as g/day. Likewise, fruit (excluding dried fruit and juice), vegetables and fish was included in units of g/day.

Finally, a diet quality index (DQI) calculated from national guidelines related to dietary intake was included to evaluate the overall quality of the children’s diet [28]. The following nutrients and food items were included in this index: fat (maximum 30 E%), saturated fat (maximum 10 E%), added sugar (maximum 10 E%), fish (minimum 200 g/week), fruit and vegetables (minimum 300 g/d) and potatoes, rice or pasta (minimum 200 g/d). For each child, a DQI score was calculated based on the six nutrients/food groups, as a function of recommended vs. reported intake. For the food groups with a minimum recommended intake, the group score was based on the ratio (R/R_T_) between the reported (R) and the recommended (R_T_) intake, where the score was set to 1 for intakes with R ≥R_T_. For the food groups with a maximum recommended intake, the score was derived as 1- (R -R_T_) /(R_max_ -R_T_), where the score was set to 1 for intakes R≤R_T_. Hence, the score for each of the food items and nutrients ranged between 0 and 1, were 1 corresponds to a score fully complying with the recommendation. From this information, a total score (ranging from 0 to 6) was generated by summing the six individual scores.

### Covariates

The children’s heights were measured to the nearest 0.1 cm using a stature meter (Soehnle 5002 or Charter ch200P), and their weights were measured to the nearest 0.1 kg using a mechanical weight or beam-scale type weight (Tanita BWB-800 or SV-SECA 710) by the health counsellors. The children were measured in underwear only and were asked to urinate before the weighing. If the child was using diaper, a new diaper was put on before the weighing. From this information we calculated BMI z-scores for the children using the Lambda-Mu-Sigma method, which summarizes the changing distributions of BMI by the median, the coefficient of variation and skew expressed as Box-Cox power [14]. The parents were also asked whether their child liked to be physically active. For the present study, we included physical activity in four categories: 1) the child never or rarely thinks it is fun being physically active, 2) the child sometimes thinks it is fun to be physically active, 3) the child usually thinks it is fun to be physically active, and 4) the child always thinks it is fun to be physically active. We also included information on intervention status (intervention/control), gender and age (years). Moreover, the parents reported their own weight and height, from which BMI was calculated by dividing weight in kg with height in meters squared (kg/m2). The parents also reported highest level of completed education, which was included in the analyses in four categories: 1) low education level (including “primary and lower secondary”, “upper secondary”, “one or more short courses” and “skilled worker”), 2) medium education level (including “short-term further education [<3 years]” or “medium-term further education [3–4 years]”), 3) high education level (including “long-term further education [>4 years]”, “research level”) and 4) other educations (including educational information which was not possible to classify according to above [e.g. education completed in foreign countries]).

### Statistical analyses

Linear regression analyses, with bootstrapped confidence intervals (CI), were conducted to access the association between HCC and dietary variables. First, crude models with HCC measures and information on dietary intake were conducted. Secondly, adjusted analyses, with added information on age, gender, BMI Z-score, physical activity, intervention status and maternal education, were conducted. We adjusted for maternal rather than paternal education as the mother in most cases was the primary caretaker. Moreover, studies have found that low maternal education influences different aspects of offspring dietary intake [29,30] and predicts childhood obesity [31]. The same set of analyses was also conducted for maternal and paternal HCC, following the same adjustment scheme, but with added information on parental BMI and paternal education in analyses of the fathers HCC.

Gender and/or intervention-status interactions were tested by adding product terms to the models and stratified analyses were conducted when statistically significant interactions were observed.

Moreover, an association between maternal HCC and child HCC has previously been shown [32]. Thus, as sensitivity analyses, we further adjusted the analyses of child HCC for parental HCC, and the analyses of parental HCC for child HCC.

All statistical tests were two-sided with a significance level at 0.05. Analyses were performed using Stata SE 14 (StataCorp LP, College Station, Texas, USA; www.stata.com).

## Results

Information on HCC, dietary intake and covariates are shown in Table 1. The median HCC was 92 pg/mg (5–95 percentiles: 23–416) among the children, 122 pg/mg (5–95 percentiles 47–329) among the mothers, and 148 pg/mg (5–95 percentiles: 52–404) among the fathers.

**Table 1:** Study characteristics of children and parents. Results presented as median and 5–95 percentiles unless stated otherwise.

	Children	Mothers	Fathers
**n**	296	272	208
**Age (years)**	5.5 (3.7, 7.0)	-	-
**Gender (% girls)**	44	-	-
**HCC (pg/mg)**	92 (23, 416)	122 (47, 329)	148 (52, 404)
**Total energy (kcal/day)**	1284 (890, 1776)	-	-
**Total fat (g/day)**	45 (27, 67)	-	-
**Total fat (E%)**	30 (22, 39)	-	-
**Protein (g/day)**	50 (31, 73)	-	-
**Protein (E%)**	15 (12, 20)	-	-
**Carbohydrate (g/day)**	181 (116, 250)	-	-
**Carbohydrate (E%)**	54 (45, 63)	-	-
**Added sugar (g/day)**	20 (6, 53)	-	-
**Fruit and vegetables (g/day)**	202 (65, 408)	-	-
**SSB (g/day)**	50 (0, 256)	-	-
**DQI (0–6)**	4.3 (3.4, 5.3)	-	-
**BMI Z-score (SD)**	0.3 (-1.1, 1.8)	-	-
**Intervention group (%)**	42	42	45
**PA (% most active)**	39	-	-
**Parental education level (% low)**	-	18	33
**Parental BMI**	-	25.2 (20.2, 36.8)	25.7 (21.3, 32.9)

Abbreviations: HCC, hair cortisol concentration; SSB, sugar-sweetened beverages; DQI, diet quality index; BMI, body mass index; PA, physical activity

### Total energy intake and macronutrients

The associations between child and parental HCC, total energy and macronutrients among the children are shown in Table 2. For the children, higher HCC was associated with a lower fat consumption (adjusted β: -0.7 g/day [95% CI: -1.3, -0.0, P = 0.04] per 100 pg/mg HCC). We found no evidence of association between child HCC and total energy intake, protein or carbohydrate.

**Table 2:** Association between child and parental hair cortisol concentration (units of 100 pg/mg) and macronutrient intake among children in the Healthy Start study.

		Total energy		Fat				Protein				Carbohydrate			
	n	kcal/day	P	g/day	P	E%	P	g/day	P	E%	P	g/day	P	E%	P
**Child HCC**															
Crude	296	-14 (-29, 1)	0.07	-0.8 (-0.2, 0.0)	0.05	-0.2 (-0.5, 0.1)	0.14	-0.5 (-1.3, 0.3)	0.24	0.0 (-0.2, 0.2)	0.86	-1.3 (-3.4, 0.9)	0.24	0.2 (-0.1, 0.0)	0.20
Adjusted^1^	296	-11 (-25, 3)	0.13	-0.7 (-1.3, -0.0)	0.04	-0.2 (-0.5, 0.1)	0.19	-0.4 (-1.2, 0.3)	0.22	0.0 (-0.2, 0.1)	0.92	-0.8 (-3.6, 1.8)	0.52	0.2 (-0.1, 0.0)	0.21
**Maternal HCC**															
Crude	272	-22 (-62, 18)	0.27	-1.0 (-2.4, 0.4)	0.17	-0.1 (-0.7, 0.4)	0.66	-0.6 (-2.3, 1.1)	0.50	0.1 (-0.3, 0.5)	0.68	-2.8 (-7.9, 2.3)	0.28	0.0 (-0.6, 0.7)	0.92
Adjusted^2^	272	0 (-40, 40)	0.99	-0.1 (-1.9, 1.6)	0.87	0.0 (-0.7, 0.6)	0.89	0.1 (-1.5, 1.8)	0.86	0.0 (-0.3, 0.4)	0.86	0.2 (-5.9, 6.2)	0.96	0.0 (-0.7, 0.7)	0.96
**Paternal HCC**															
Crude	208	-11 (-42, 20)	0.50	-0.3 (-1.7, 1.0)	0.64	0.0 (-0.4, 0.5)	0.83	-0.7 (-2.4, 1.0)	0.40	-0.1 (-0.4, 0.2)	0.40	-1.5 (-6.4, 3.5)	0.56	0.1 (-0.4, 0.5)	0.79
Adjusted^3^	208	-11 (-42, 20)	0.48	-0.4 (-2.2, 1.4)	0.65	0.0 (-0.7, 0.7)	0.99	-0.1 (-2.3, 0.1)	0.07	-0.2 (-0.5, 0.0)	0.07	-1.0 (-4.7, 2.8)	0.62	0.2 (-0.4, 0.8)	0.46

Results presented as β (in units of 100 pg/mg) and corresponding 95% CIs

Abbreviations: HCC, hair cortisol concentration; BMI, body mass index; PA, physical activity

^1^ Adjusted for age, gender, BMI Z-score, PA, intervention status and maternal education

^2^ Adjusted for age, gender, BMI Z-score, PA, intervention status, maternal education and maternal BMI

^3^ Adjusted for age, gender, BMI Z-score, PA, intervention status, paternal education and paternal BMI

Moreover, no association between maternal or paternal HCC and intake of total energy, fat, protein or carbohydrate among their children was found. Likewise, no association between child or parental HCC and percent of energy from fat, protein or carbohydrate was found.

We found no evidence of interaction between child or maternal HCC and the child’s gender in relation to intake of energy or macronutrients. However, we found an interaction between paternal HCC and child gender in relation to the intake of total energy (P = 0.03) and protein (P = 0.02). Stratified analyses showed that paternal HCC was associated with a borderline significant lower total energy and significantly lower protein intake among their daughters (adjusted β: -42 kcal/day [95% CI: -85, 0, P = 0.052] and -2.6 g/day [95% CI: -4.4, -0.8, P = 0.01] per 100 pg/mg HCC, respectively) but not among sons (adjusted β: 12 kcal/day [95% CI: -32, 56, P = 0.60] and -0.1 g/day [95% CI: -1.9, 1.7, P = 0.92] per 100 pg/mg HCC, respectively). Finally, we found no evidence of interactions between HCC and intervention status in any of the analyses.

### Added sugar, selected food groups and diet quality

We found no evidence of association with added sugar, fruit and vegetables, SSB or DQI in analyses of child, maternal or paternal HCC (Table 3). Finally, we found no evidence of interactions between HCC and gender or intervention status in any of these analyses.

**Table 3:** Association between child and parental hair cortisol concentration (units of 100 pg/mg) and added sugar, selected food groups and diet quality among children in the Healthy Start study.

		Added sugar		Fruit & vegetables		SSB		DQI	
	N	g/day	P	g/day	P	g/day	P	score	P
**Child HCC**									
Crude	296	-0.4 (-1.2, 0.5)	0.37	1.2 (-5.9, 8.3)	0.74	3.3 (-5.3, 11.9)	0.45	-0.0 (-0.0, 0.0)	0.96
Adjusted^1^	296	0 (-1.1, 1.0)	0.93	0.4 (-5.7, 5.0)	0.90	4.8 (-4.3, 13.8)	0.30	0.0 (-0.0, 0.0)	0.65
**Maternal HCC**									
Crude	272	-1.2 (-4.3, 1.9)	0.46	-8.9 (-2.3, 5.4)	0.22	3.3 (-12.3, 19.0)	0.45	-0.0 (-0.1, 0.0)	0.44
Adjusted^2^	272	-0.7 (-3.2, 1.7)	0.54	-8.8 (-2.1, 3.5)	0.16	4.4 (-7.9, 16.7)	0.48	-0.0 (-0.1, 0.0)	0.39
**Paternal HCC**									
Crude	208	0.2 (-1.3, 1.7)	0.79	-5.6 (-1.9, 8.5)	0.43	7.4 (-4.6, 19.5)	0.23	-0.0 (-0.1, 0.0)	0.35
Adjusted^3^	208	0.1 (-1.7, 1.8)	0.95	-5.3 (-1.9, 8.2)	0.44	7.6 (-4.3, 19.5)	0.21	-0.0 (-0.1, 0.0)	0.35

Results presented as β (in units of 100 pg/mg) and corresponding 95% Cis

HCC, hair cortisol concentration; SSB, sugar-sweetened beverages; DQI, diet quality index; BMI, body mass index; PA, physical activity

^1^ Adjusted for age, gender, BMI Z-score, PA, intervention status and maternal education

^2^ Adjusted for age, gender, BMI Z-score, PA, intervention status, maternal education and maternal BMI

^3^ Adjusted for age, gender, BMI Z-score, PA, intervention status, paternal education and paternal BMI

### Sensitivity analyses

Further adjustment for parental HCC, when analysing the associations between child HCC and dietary intake, did not change the observed results, nor did adjustment for child HCC affect the associations between parental HCC and child dietary intake (S1 and S2 Tables).

## Discussion

In this cross-sectional study of primarily normal-weight preschool children predisposed to overweight and obesity, we found that higher HCC was associated with a lower consumption of dietary fat, while no statistically significant associations were found between HCC and intake of total energy, protein, carbohydrate, fruit and vegetables, added sugar, SSB or DQI was found. Moreover, we found no evidence of association between parental HCC and intake of total energy, added sugar, selected food groups or DQI among their children. However, stratified analyses showed that paternal HCC was associated with a borderline significant lower total energy intake and a significantly lower protein intake among their daughters but not among their sons.

We found no previous studies that had investigated the relationship between HCC and dietary intake. However, our results are in contrast to observations by Michels et al. (2013), showing that higher cortisol concentrations were associated with higher intakes of both fat and sugar among 5–10 year old children, when using salivary rather than hair cortisol as a biomarker of stress [13]. Likewise, our results are inconsistent with the results by George et al. (2010), showing that elevated cortisol after infusion of corticotropin-releasing hormone predicted a higher subsequent calorie intake among adults [12]. Our results also suggested some gender difference, as paternal HCC was associated with a borderline significant lower total energy and a significantly lower protein intake among their daughters but not among their sons. It has been suggested that girls are more sensitive to parental stress than boys [33], which may explain the observed gender difference, though this remains speculative. As we found no previous studies suggesting a similar gender specific relationship between paternal stress (cortisol secretion or psychosocial stress) and dietary intake among their children, these results need replication in future studies. Moreover, although our study suggests some statistically significant association it is worth mentioning that the observed association were extremely weak. For example, we found that each 100 pg/mg higher paternal HCC, which is approximately 68% of the median HCC for the fathers, was associated with 2.6 g/day lower protein intake among daughters, only.

It has been hypothesized that the relationship between elevated cortisol and increased consumption of total calories, fat and sugar are stimulated by reward centre activation through cortisol-induced opioid increases and perhaps indirectly through cortisol-induced increase in insulin and leptin [13]. However, our findings indicate that these biological mechanisms may only apply to acutely increased cortisol, while chronically elevated cortisol, as measured by HCC, may affect appetite and cravings in a different way, or that an effect of cortisol on the reward system decreases after prolonged exposure. Moreover, our study suggests no or limited mediating influence of dietary intake on the relationship between HCC and adiposity observed in other studies (15–22), and on the association between maternal HCC and the child’s body composition observed in a previous publication based on the Healthy Start study (13).

Our study has a number of strengths, including data on HCC, a validated biomarker of long-term cortisol exposure [18,34,35], on a relatively large group of children and parents, detailed information on dietary intake, in addition to questionnaire information on several other lifestyle factors, allowing us to adjust for potential confounders.

Nevertheless, the study also has some limitations. Although HCC reflect the average stress level in the months prior to the time-point dietary data was collected, our analyses were basically cross-sectional, and as a result we cannot establish causality. Moreover, HCC was measured at follow-up, and thus it is possible that the intervention itself had affected the associations. However, as we found no evidence of on interaction between HCC and intervention status in any of the analyses, this seems unlikely. In addition, while we had information on a large sample of children and parents, we cannot rule out that we have overlooked associations as a result of insufficient statistical power, although the generally narrow CIs would suggest that it is unlikely we overlooked any noteworthy associations. Moreover, regardless of the method used to collect dietary data, these methods are generally subject to inaccuracies. Hence, measurement error related to dietary intake could have biased the results towards null and led to wider confidence intervals. In addition, people who agree to participate in trials are likely to eat healthier and be less stressed than the background population. Thus, the variance in stress and dietary intake might not have been sufficiently large to find associations. In support of this, previous results from the Healthy Start study suggest that the enrolled participants generally ate according to the Danish national recommendations, with quite limited variation in intake of the selected nutrients and foods [28]. We can also not rule out differential misclassification, as the stressed parents potentially may have had a tendency to provide a less adequate dietary registration for their children than the less stressed parents, which could have attenuated a positive relationship. We also relied on self-reported information on several variables. This may be problematic in some cases. For example, misreporting of parental weight and height is possible. Lastly, as in most observational studies, unknown and residual confounding may be present although we adjusted for several likely cofounding factors. HCC is a relatively new biomarker and although several determinants have been identified and included in the analyses, we cannot rule out confounding from unknown or unmeasured variables. Nonetheless, according to a recent systematic review on determinants of hair cortisol among children, the most important confounding factors to consider are gender and anthropometry [23], factors that were adjusted for in our analyses.

The present study is based on data from the follow-up of the Healthy Start children. Thus, due to loss to follow-up and missing data, we do not have information on all participants enrolled in the study (47%). Moreover, our results originate from the specific group of primarily normal weight children who were predisposed to obesity, and thus generalization to all children of similar age needs to be done with caution. Nevertheless, we see no obvious reason why the results should be different in other populations, since it is the influence of cortisol on dietary intake which should be representative, and not necessarily the examined population as a whole.

In conclusion, our study suggests that chronic stress as measured by HCC may be associated with a lower fat consumption among children, and that paternal HCC may be associated with a lower intake of energy and protein among their daughters. However, the associations observed were weak, and any clinical relevance of these findings remains questionable.

## Supporting information

S1 Fig Study flowchart. (DOCX)

S2 Fig Number of participants from each inclusion criteria. (DOCX)

S1 Table Association between child and parental hair cortisol concentration (units of 100 pg/mg) and macronutrient intake among children (with additional adjustment for parental or child hair cortisol concentration). (DOCX)

S2 Table Association between child and parental hair cortisol concentration (units of 100 pg/mg) and added sugar, selected food groups and diet quality among children (with additional adjustment for parental or child hair cortisol concentration). (DOCX)
