# Supplementary material for: Association between hair cortisol concentration and dietary intake among normal weight preschool children predisposed to overweight and obesity
Source: PLoS One. 2019 Mar 8;14(3):e0213573. doi: 10.1371/journal.pone.0213573 (PMC6407774; doi:10.1371/journal.pone.0213573)
Supplement: S1 Fig — (DOCX) [file pone.0213573.s001.docx]

**S1 Fig:** **Study flowchart**


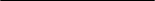

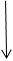


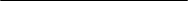


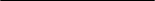

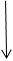

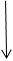

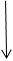

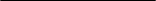


Did not participate

**n 2667**

Examination

**n 635**

**n 320**

Intervention group:

**n 315**

Control group:

*Excluded*

Children with missing information on dietary intake, hair cortisol and covariates at follow-up: **n 339**

Study population with complete

Information at follow-up

**n 296**

Intervention group: **n 123**

Control group: **n 173**

Children participating at baseline

Study participants at baseline

**n 1055**

Intervention group:

**n 320**

Control group:

**n 315**

Shadow control group: **n 420**
