# Supplementary material for: Association between hair cortisol concentration and dietary intake among normal weight preschool children predisposed to overweight and obesity
Source: PLoS One. 2019 Mar 8;14(3):e0213573. doi: 10.1371/journal.pone.0213573 (PMC6407774; doi:10.1371/journal.pone.0213573)
Supplement: S2 Fig — (DOCX) [file pone.0213573.s002.docx]

**S2 Fig: Number of participants from each inclusion criteria**

n = 77

n = 3

n = 2

n = 0

Maternal overweight prior to pregnancy

Maternal low SES at birth

High birth weight

n = 189

n = 24

n = 1

|  |  |  |  |  |
| --- | --- | --- | --- | --- |
|  |  |  |  |  |
|  |  |  |  |  |
|  |  |  |  |  |
