# Supplementary material for: Association between hair cortisol concentration and dietary intake among normal weight preschool children predisposed to overweight and obesity
Source: PLoS One. 2019 Mar 8;14(3):e0213573. doi: 10.1371/journal.pone.0213573 (PMC6407774; doi:10.1371/journal.pone.0213573)
Supplement: S1 Table — (DOCX) [file pone.0213573.s003.docx]

| **S1 Table: Association between child and parental hair cortisol concentration (units of 100 pg/mg) and macronutrient intake among children (with additional adjustment for parental or child hair cortisol concentration)** | | | | | | | | | | | | | | | |
| --- | --- | --- | --- | --- | --- | --- | --- | --- | --- | --- | --- | --- | --- | --- | --- |
|  |  | **Total energy** | | **Fat** | | | | **Protein** | | | | **Carbohydrate** | | | |
|  | **n** | **kcal/day** | **P** | **g/day** | **P** | **E%** | **P** | **g/day** | **P** | **E%** | **P** | **g/day** | **P** | **E%** | **P** |
| **Child HCC** | | | | | | | | | | | | | | | |
| Adjusted^1^ | 193 | -13  (-36, 1) | 0.24 | -0.9  (-1.7, -0.0) | 0.03 | -0.3  (-0.5, 0.0) | 0.05 | -0.4  (-1.4, 0.5) | 0.39 | 0.0  (-0.2, 0.2) | 0.70 | -1.0  (-4.6, 2.6) | 0.58 | 0.2  (-0.1, 0.6) | 0.23 |
| **Maternal HCC** | | | | | | | | | | | | | | | |
| Adjusted^2^ | 264 | 0  (-31, 37) | 0.88 | -0.0  (-1.5, 1.5) | 0.99 | -0.0  (-0.6, 0.6) | 0.95 | 0.2  (-2.0, 2.4) | 0.87 | 0.0  (-0.4, 0.4) | 0.97 | 0.5  (-3.6, 4.6) | 0.81 | 0.0  (-0.6, 0.6) | 0.98 |
| **Paternal HCC** | | | | | | | | | | | | | | | |
| Adjusted^3^ | 203 | -0  (-36, 36) | 0.99 | 0.3  (-1.6, 2.1) | 0.77 | 0.2  (-0.4, 0.8) | 0.49 | -0.6  (-2.3, 1.1) | 0.48 | -0.2  (-0.6, 0.1) | 0.22 | -0.1  (-4.4, 4.1) | 0.94 | 0.0  (-0.6, 0.6) | 0.99 |
| *Results presented as β (in units of 100 pg/mg) and corresponding 95% CIs* | | | | | | | | | | | | | | | |
| *Abbreviations: HCC, hair cortisol concentration; BMI, body mass index; PA, physical activity* | | | | | | | | | | | | | | | |
| *^1^Adjusted for age, gender, BMI Z-score, PA, intervention status, maternal education and parental hair cortisol concentration* | | | | | | | | | | | | | | | |
| *^2^Adjusted for age, gender, BMI Z-score, PA, intervention status, maternal education, maternal BMI and child hair cortisol concentration* | | | | | | | | | | | | | | | |
| *^3^Adjusted for age, gender, BMI Z-score, PA, intervention status, paternal education, paternal BMI and child hair cortisol concentration* | | | | | | | | | | | | | | | |
