# Supplementary material for: Association between hair cortisol concentration and dietary intake among normal weight preschool children predisposed to overweight and obesity
Source: PLoS One. 2019 Mar 8;14(3):e0213573. doi: 10.1371/journal.pone.0213573 (PMC6407774; doi:10.1371/journal.pone.0213573)
Supplement: S2 Table — (DOCX) [file pone.0213573.s004.docx]

| **S2 Table: Association between child and parental hair cortisol concentration (units of 100 pg/mg) and added sugar, selected food groups and diet quality among children (with additional adjustment for parental or child hair cortisol concentration)** | | | | | | | | | |
| --- | --- | --- | --- | --- | --- | --- | --- | --- | --- |
|  |  | **Added sugar** | | **Fruit & vegetables** | | **SSB** | | **DQI** | |
|  | **n** | **g/day** | **P** | **g/day** | **P** | **g/day** | **P** | **score** | **P** |
| **Child HCC** | | | | | | | | | |
| Adjusted^1^ | 193 | 0.0  (-1.7, 1.9) | 0.93 | 1.3  (-7.2, 9.7) | 0.77 | 4.6  (-9.9, 19.2) | 0.53 | 0.0  (-0.1, 0.1) | 0.87 |
| **Maternal HCC** | | | | | | | | | |
| Adjusted^2^ | 264 | -0.8  (-3.4, 1.9) | 0.57 | -8.9  (-2.1, 3.4) | 0.16 | 3.1  (-11.2, 17.4) | 0.67 | -0.0  (-0.1, 0.0) | 0.41 |
| **Paternal HCC** | | | | | | | | | |
| Adjusted^2^ | 203 | 0.2  (-1.8, 2.2) | 0.84 | -5.0  (-1.7, 7.1) | 0.42 | 5.8  (-8.2, 19.8) | 0.41 | -0.0  (-0.1, 0.0) | 0.50 |
| *Results presented as β (in units of 100 pg/mg) and corresponding 95% Cis* | | | | | | | | | |
| *HCC, hair cortisol concentration; SSB, sugar-sweetened beverages; DQI, diet quality index; BMI, body mass index; PA, physical activity* | | | | | | | | | |
| *^1^Adjusted for age, gender, BMI Z-score, PA, intervention status, maternal education and parental hair cortisol concentration* | | | | | | | | | |
| *^2^Adjusted for age, gender, BMI Z-score, PA, intervention status, maternal education, maternal BMI and child hair cortisol concentration* | | | | | | | | | |
| *^3^Adjusted for age, gender, BMI Z-score, PA, intervention status, paternal education, paternal BMI and child hair cortisol concentration* | | | | | | | | | |
